# Predation evaluation of the green lacewing, *Chrysopa pallens* on the pink tea mite pest, *Acaphylla theae* (Watt) (*Acarina*: *Eriophyidae*)

**DOI:** 10.3389/fphys.2023.1307579

**Published:** 2023-12-12

**Authors:** Qian Wang, Meng Zhang, Qiuyu Guo, Chenxin Wu, Liang Sun

**Affiliations:** ^1^ The Key Laboratory for Quality Improvement of Agricultural Products of Zhejiang Province, College of Advanced Agricultural Sciences, Zhejiang A & F University, Hangzhou, China; ^2^ Key Laboratory of Tea Quality and Safety Control, Ministry of Agriculture and Rural Affairs, Tea Research Institute, Chinese Academy of Agricultural Sciences, Hangzhou, China

**Keywords:** *Acaphylla theae*, *Chrysopa pallens*, DNA-based gut content analyses, predator-prey interactions, functional responses

## Abstract

A better understanding of predator-prey interactions is crucial for the development of biological control strategies. The green lacewing, *Chrysopa pallens*, is a well-known generalist predator and reportedly functions as one of the most important biological control agents of insect pests. However, information regarding *C*. *pallens*’ predation on tea plant pests, particularly notorious tea mites, remains largely unknown. In this study, we focused on the predator-prey relationship between *C*. *pallens* and an important tea mite pest, *Acaphylla theae*. We designed species-specific primers for the detection of *A. theae* DNA and established a PCR-based DNA gut content analysis assay. These results demonstrated that the primers were *A. theae*-specific and suitable for its molecular identification. The laboratory feeding experiment showed that the detectability success (DS_50_) of *A. theae* DNA remaining in *C. pallens’* guts was 2.9 h. We then performed a molecular detection of field predation, and achieved a 23.53% positive detection rate of *A. theae* DNA in the guts of field-collected *C. pallens*. This, for the first time, provides direct evidence that *C. pallens* can prey on *A. theae* in tea plantations. Finally, we tested the prey preference and estimated the predation ability of *C. pallens* on different developmental stages of *A. theae*. The results revealed that *C. pallens* had no significant preference for different developmental stages of *A. theae*. The functional responses of *C. pallens*’ predation on different densities of *A. theae* at different developmental stages followed a Type II Holling model. The initial attack rate (a’) ranged from 0.735 to 0.858 and the handling time (T_h_) was approximately 0.01. This study is the first to demonstrate the trophic interactions between *C. pallens* and *A. theae* and provides evidence for the development of biological control strategies against *A. theae* using *C. pallens* as a candidate predator.

## Introduction

Tea contains characteristic secondary metabolites (Liao et al., 2022), including catechin, theanine, and caffeine, which have numerous health benefits for humans and is, therefore, considered the most popular nonalcoholic beverage consumed worldwide (Yang et al., 2009). The tea plant *Camellia sinensis* (L.) O. Kuntze is one of the most economically important agricultural crops worldwide. In China, tea cultivation and production play important roles in poverty alleviation and rural revitalization (Ye et al., 2021). Therefore, the protection of tea plants against serious insect and mite pests have crucial economic and social significance.

It has been reported that approximately 800 species of insect and mite pests, belonging to 109 families from 13 orders, have been recorded in Chinese tea plantations; of which the pink tea mite pest, *Acaphylla theae* (Watt) (Acarina: Eriophyidae), is one of the most serious (Ye et al., 2014). *Acaphylla theae* is a global mite pest threatening tea production in China and other countries. It destroys tea plants by sucking the leaves and causing severe damage; including leaf luster loss, bud leaf atrophy, rust spots of different colors, brittle cracking, and defoliation (Li et al., 2021).

*Acaphylla theae* is difficult to control owing to its small body size, high concealment of symptoms, short reproductive cycle, and strong adaptability (Yin et al., 2003). Unlike most amphigenetic insect pests, such as hemipteran bugs and lepidopteran moths, where eco-friendly pest control can benefit from sex pheromone identification and molecular basis analyses (He et al., 2017; He et al., 2020; Wang et al., 2020; Yan et al., 2020; Wang et al., 2022a; Zhang et al., 2023), *A. theae* belongs to parthenogenetic mite species and it is impossible to develop eco-friendly management through sexual trapping. Although multiple attempts have been made to develop plant-sourced acaricides (Tong and Feng, 2016) and identify highly resistant tea plants (Yao et al., 2008), *A. theae* control is mainly dependent on the application of pesticides which results in the rapid development of mite resistance, undesirable residues on tea, and damage to non-target beneficial arthropods (Li et al., 2021). Highly efficient and eco-friendly management of *A. theae* is urgently required to improve tea quality and safety.

Biological control through the utilization of natural enemies has fewer negative effects on the environment and could be an alternative pest management strategy to traditional methods (Kitto, 1983). In China, over 1,100 species of natural enemies have been identified in tea plantations; with arthropod predators being the most abundant, accounting for 54.5% of the total number of species (Ye et al., 2014). Despite the abundant variety of natural enemies of tea plantations, native natural enemies are not available as candidate predators of *A. theae*. This could be partially attributed to the difficulty in accurately identifying predator-prey interactions using conventional methods, especially in the field. Gut DNA content analysis could be an excellent alternative strategy because it can specifically identify minute amounts of target DNA from insect pest diets, and this approach has become the most widely used for identifying predator-prey interactions (Symondson, 2002; King et al., 2008; Greenstone et al., 2014).

The green lacewing *Chrysopa pallens* (Rambur) (Neuroptera: Chrysopidae) is a generalist predator (Lai and Liu, 2020). It has been extensively studied for its biological control potential for development of sustainable and integrated pest management strategies to reduce the application of chemical pesticides in forests and agricultural ecosystems. Several studies have reported predation by *C. pallens* on various insect pests, including thrips (Sarkar et al., 2019), aphids (Zhao et al., 2008; Sun et al., 2013), whiteflies (Liu et al., 2011; Tang et al., 2018), and the young larvae and eggs of Lepidoptera (Canard and Volkovich, 2001; Cao et al., 2020). In addition, great progress has been made in the understanding of *C. pallens*’ biology and ecology (Kunkel and Cottrell, 2007; Wang et al., 2022b), development (Guo et al., 2008), factors affecting its predation ability (Abdrabou, 2008; Wang et al., 2021), and the toxicity and sublethal effects of insecticides (Su et al., 2022). Despite *C. pallens* being one of the most common predators, whether it preys on mite pests in tea gardens and the predator–prey interactions between *C. pallens* and *A. theae* remain largely unclear.

In this study, we designed species-specific primers for *A. theae* and established a PCR-based assay to identify prey DNA remaining in the *C. pallens*’ guts. We then used this assay to test the predator-prey relationship between field-collected *C. pallens* and *A. theae*. Finally, we determined the feeding preferences and functional responses of *C. pallens* to *A. theae* at different developmental stages. These results help determine how *C. pallens* preys on *A. theae* and provide a foundation for the development of biological control management strategies against *A. theae* in tea gardens.

## Materials and methods

### Mite and insect colonies

*Acaphylla theae* were collected from tea plantations at Zhejiang A & F University (119.7°E, 30.2°N). The leaves and branches of the tea trees with mites were cut, brought back to the laboratory and raised in glass bottles containing water. The number of mites was observed under the stereomicroscope. Colony establishment was obtained by transferring 50–100 mites from infested leaves and branches to a non-infested tea leaf. The colony was subsequently placed at 25°C ± 1°C, 70%–80% relative humidity, and an L16: D8 photoperiod.

*Chrysopa pallens* colonies were established in the laboratory at the Zhejiang A & F University. All larvae and adults were reared on cowpea aphid *Aphis craccivora* Koch (Hemiptera: Aphididae) in 20 × 13 × 8.5 cm transparent plastic rearing containers with a nylon screen top at 24°C ± 1°C, 65%–75% relative humidity, and an L16: D8 photoperiod.

### DNA extraction

*Acaphylla theae* DNA was extracted by the Chelex extraction method (Yang et al., 2020). Ten adult *A. theae* were placed in the bottom of a 1.5 mL centrifuge tube and ground by a sterile pestle. Then, 150 μL 10% Chelex (BIO-RAD), 20 μL PBS solution (pH 7.2–7.4, Sangon Biotech, Shanghai), and 30 μL Proteinase K (20 mg/mL, Tiangen Biotech, Beijing) were added to the 1.5 mL centrifuge tube. The ground mite tissues and the reagents were briefly vortexed before being placed in a thermos metal bath (BTC-100D; MIULab, Hangzhou, China) and heated to 56°C for 8 h and 96°C for 20 min.

The whole body DNA of *C. pallens* and other invertebrate species was extracted using a FastPure^®^ Blood/Cell/Tissue/Bacteria DNA isolation Mini Kit (Nanjing Vazyme Biotech Co., Ltd., Nanjing, China), according to the manufacturer’s instructions. Two negative control samples, using PCR-grade water instead of the DNA extract, were included during the DNA extraction procedure to demonstrate that there was no contamination.

### Primer design and species-specific identification

The 28S ribosomal RNA gene was chosen as the DNA barcode for the molecular identification of *A. theae* because it has been proven to be suitable for the molecular classification and identification of mites (Zhao et al., 2020). Sequences of the 28S rRNA gene region of *A. theae* were obtained from GenBank (KJ145939). The 28S rRNA genes of *A. theae* and other non-target arthropods were aligned using the BioEdit sequence alignment editor 7.1.3.0 (Hall, 1999) and the specific primers (Table 1) were designed using Primer Premier 5 version 5.00 (Lalitha, 2000). The specificity of the primers was tested on 38 non-target invertebrate species belonging to 26 different families (Table 2) that had been starved for 2 days or 7 days (Traugott et al., 2013; Li et al., 2017).

**TABLE 1:** Primer sequences and amplicon size of *A. theae*.

Primer name	Sequence (5′-3′)	Annealing T (°C)	Size (bp)
*Athe28S rRNA-D2F*	CGC​AAG​ATT​TTG​GGT​GTA​TTC​TT	59	136
*Athe28S rRNA-D2R*	TGGCCCAAGCGGACAATA		

**TABLE 2:** Invertebrate species used for the primers’ specificity test.

Class	Order	Family	Species
Insecta	Hemiptera	Aphididae	*Toxoptera aurantii* (Boyer de Fonsco10mbe)
			*Aphis craccivora* Koch
		Cicadellidae	*Empoasca onukii* (Matsuda)
		Miridae	*Apolygus lucorμm* (Meyer-Dür)
		Pentatomidae	*Halyomorpha halys* (Stål)
		Lygaeidae	*Geocoris pallidipenn*is (Costa)
		Aleyrodidae	*Aleurocanthus spiniferus* (Quaintance)
		Coccidae	*Ceroplastes japonicus* Green
		Aleyrodidae	*Aleurocanthus spiniferus* (Quaintanca)
	Thysanoptera	Thripidae	*Dendrothrips minowai* Priesner
	Coleoptera	Coccinellidae	*Harmonia axyridis* (Pallas)
			*Propylaea japonica* (Thunberg, 1781)
		Curculionidae	*Myllocerinus aurolineatus* Voss
	Lepidoptera	Geometridae	*Ectropis obliqua* Prout
			*Ectropis grisescen*s Warren
			*Scopula subpunctaria* (Herrich-Schaeffer)
			*Jankowskia athlete* Oberthur
		Lymantriidae	*Euproctis pseudoconspersa* Strand
		Gracilariidae	*Caloptilia theivora* (Walsingham)
		Limacodidae	*Thosea sinensis* Walker
	Neuroptera	Chrysopidae	*Chrysoperla sinica* (Tjeder)
			*Chrysopa pallens* (Rambur)
	Diptera	Syrphidae	*Episyrphus balteatus* (De Geer)
			*Eupeodes corollae* Fabricius
Arachnida	Araneae	Salticidae	*Evarcha albaria* (Koch, 1878)
			*Plexippus setipet* Karsch, 1879
			*Phintella bifurcilinea* (Boes. et Str., 1906)
			*Carrhotus xanthogramma*
			*Phintella yinae*
		Globulidae	*Coleosoma octomaculatum*
		Araneidae	*Araneus ejusmodi* Boes. et Str., 1906
			*Neoscona theis*i (Walckenaer, 1842)
		Aracidae	*Misumenops tricuspidatus*
		Agelenidae	*Agelena labyrinthica* Clerck, 1758
		Clubionidae	*Clubiona reichlini* Schenkel, 1944
		Thomisidae	*Xysticus ephippiatus* (Simon)
		Oxyopidae	*Oxyopes sertatus* L. Koch, 1877
		Sioscardidae	*Tetragnatha maxillosa* (Thoren, 1895)
	Trombidiformes	Eriophyidae	*Acaphylla theae* Watt

### PCR and electrophoresis

PCR was performed in 20 µL reactions containing 1 µL DNA extract, 2 µL 10 × Taq buffer (TransGen Biotech, Beijing, China), 0.4 µL dNTP, 0.2 µL Easy Taq (TransGen Biotech, Beijing, China), 0.75 µL each primer (10 µM), and 14.9 µL autoclaved, distilled water. For each PCR assay, two positive *A. theae* DNA samples were used to determine that the amplification was successful; and two negative controls, PCR-grade water instead of extracted insect DNA, were used to ensure that there was no DNA carryover contamination. PCR was performed in a Veriti 96-Well Thermal Cyclers (Applied Biosystems, United States). The amplification protocol was as follows: 95°C for 10 min, followed by 35 cycles of 95°C for 30 s, 58°C for 30 s, and 72°C for 1 min, followed by a final extension of 72°C for 10 min. The 6 µL PCR products were then separated on a 2% agarose gel and visualized under a UV transilluminator.

### Prey DNA detectability half-life determination

A feeding experiment was performed to determine the detectability of ingested *A. theae* DNA in *C. pallens* at different time points post feeding. Before the feeding trials, all the *C. pallens* predators were starved for 24 h at 25°C ± 1°C. Each four-day-old adult *C. pallens* predator was provided with 40 adult *A. theae* in glass bottles (10 cm diameter and 4 cm height) and observed under a stereomicroscope every 10 min for 1 h to ensure that they preyed on the mites. After feeding, individual predator *C. pallens* were transferred to new tubes and maintained at 25°C for 0, 4, 8, 12, 16, or 20 h (10 individuals per time point, and three repetitions per time treatment), after which they were frozen at—20°C. The DNA of *C. pallens* was extracted, and *A. theae* DNA in *C. pallens*’ guts was detected using the *A. theae*-specific PCR assay described above.

### Field sampling test for prey DNA in *C. pallens*’ guts

To confirm the predation of *C. pallens* on *A. theae*, *C. pallens* was collected from a tea garden and tested to determine whether *A. theae* DNA could be detected. The study was conducted in a Zhejiang A & F University tea plantation located in Hangzhou City, Zhejiang Province, China (119.7°E, 30.2°N). The tea plantation was managed according to standard agronomic practices, and insecticides were not used during the sampling period.

Lacewings were collected every 2 weeks from late April to August 2022. We selected three rows with a spacing interval greater than 15 m between them, and 30 sub-plots (2 × 2 m^2^) per row were randomly selected with a spacing interval greater than 1 m. We first collected lacewings by sweep netting (38 cm diameter) along the top of the tea canopies and then used a basin under tea canopies and beat canopies ten times with a stick. The number of lacewings was selected for further analysis. All samples were placed individually in 1.5 mL microcentrifuge tubes containing 95% ethanol and stored at −80°C until DNA extraction. Prior to DNA extraction, the body surface of the predator was rinsed twice with ddH_2_O. The DNA of field-collected predator samples was then extracted, and *A. theae* DNA in *C. pallens*’ guts was detected using the previously described *A. theae*-specific PCR assay. Two negative and two positive controls were included in the PCR amplifications.

### Developmental stage preference study

A free-choice feeding trial was conducted to evaluate whether *C. pallens* has a preference for *A. theae* at different developmental stages. The *A. theae* were supplied by introducing a piece of infested tea leaves to each dish and non-target developmental stages were removed using hair brushes. Twenty *A. theae* eggs, 20 larvae, 20 nymphs, and 20 adult mites were placed in a Petri dish (9 cm in diameter) containing fresh tea leaves, and water was provided using moistened cotton balls. One four-day-old adult predator that had been starved for 24 h was introduced in each dish and allowed to feed on the prey. The number of prey consumed at different developmental stages was recorded after 24 h using a stereomicroscope. The feeding experiment was repeated ten times.

### Functional responses study

A no-choice feeding trial was performed to determine *C. pallens*’ functional responses to *A. theae* developmental stages; including eggs, larvae, nymphs, and adults. Densities of 20, 40, 60, 80, and 120 per dish (9 cm in diameter, 5 cm high) for each prey developmental stage were tested. Tea leaves with prey of each different developmental stage were placed a petri dish (9 cm in diameter, 5 cm high), and water was provided using moistened cotton balls. A single predator, previously starved for 24 h, was placed into each dish and covered with a layer of breathable gauze. After 24 h, the number of remaining prey was recorded using a stereomicroscope. The experiment replicated 10 times per prey density at different developmental stages. Prey was placed in dishes at the aforementioned densities (20, 40, 60, 80, and 120 per dish) without any predators, serving as the control, and natural mortality rates were subsequently recorded.

### Statistical analysis

SAS 9.2 statistical software (SAS Institute, Cary, NC, United States) was used for all statistical analyses. A logistic regression (PROC GENMOD) was used in conjunction with SAS 9.2 software to assess the detectability of *A. theae* with time and estimate the DS_50_ values (Greenstone et al., 2007). The quantity of consumed eggs, larvae, nymph and adult in choice trial were compared by Chi-square tests. One-way ANOVA was employed to analyze the differences in prey consumption of different density treatments; multiple comparisons were performed using Tukey’s HSD test. The predation was estimated by matching the Holling Type II functional response model: $N_{a}=\frac{a^{\prime }NT}{1+a^{\prime }T_{h}N}$ , where *N*_*a*_ is the net prey consumption rate by the predator during selected time period, a’ is the instantaneous attack rate, *N* is the prey density, *T* is the predatory time of the predator (1 d), and *T*_*h*_ is the time required to prey on a mite of different developmental stages (handling time) (Holling, 1959; Wang et al., 2019).

## Results

### Specificity of mite prey primers

A primer pair (Table 1) amplifying the D2 region of the 28S ribosomal RNA gene was designed for the molecular identification of *A. theae*. To confirm its specificity, a PCR assay was performed to amplify the target fragment from the DNA extracted from *A. theae* and 38 non-target species (Table 2). The results showed that the expected 136 bp fragment of the 28S ribosomal RNA gene was successfully amplified from *A. theae*. No clear amplification was obtained from 38 non-target species or the negative controls (Supplementary Figure S1). These results demonstrate that the primers were species-specific and suitable for the specific molecular identification of *A. theae*.

### DNA detectability half-life

**FIGURE 1:**
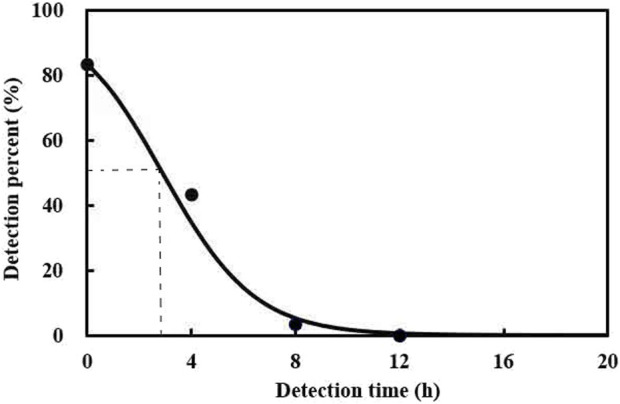
Detection of *A. theae* DNA in *C. pallens* at different times after ingestion. The model for the relationship between ingestion time (*x*) and % positive detection (*y*) was y = 100% × e^(1.6379 − 0.567x)^/(1 + e^(1.6379 − 0.567x)^), (F = 356.64, df = 2.5, *p* < 0.0001).

The prey DNA detectability half-life (DS_50_) was evaluated. The results showed that the maximum detection rate achieved for *A. theae* DNA within *C. pallens* was 83.3% and was defined as the percentage of predators exposed to *A. theae* that tested positive. After feeding, *A. theae* DNA gradually declined with increasing digestion time. The prey DNA DS_50_ of *A. theae* was estimated at 2.9 h (Figure 1).

### Molecular detection of field predation

The results of molecular detection of field predation showed that seventeen *C. pallens* adults were successfully collected in the tea field, 23.53% of which were positive for *A. theae* DNA.

### Predation preference evaluation to developmental stages

**FIGURE 2:**
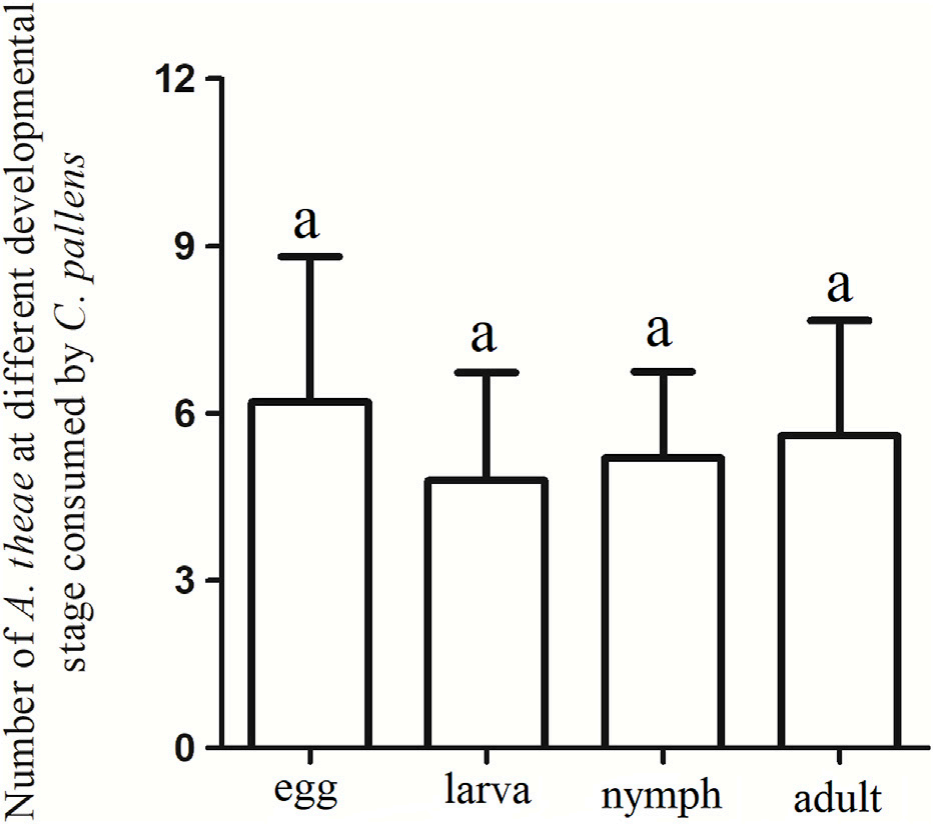
Predation preferences of *C. pallens* to *A. theae* at different developmental stages. The same letter above each bar denotes no significant differences among different treatments in the Chi-square tests.

A free-choice feeding trial was conducted to evaluate the putative predation-selective preference for *A. theae* at different developmental stages. Four-day-old *C. pallens* adults showed no feeding preference on a particular developmental stage of *A. theae*. (χ^2^ = 0.2508, df = 3, *p* = 0.969) (Figure 2).

### Predation functional responses

**FIGURE 3:**
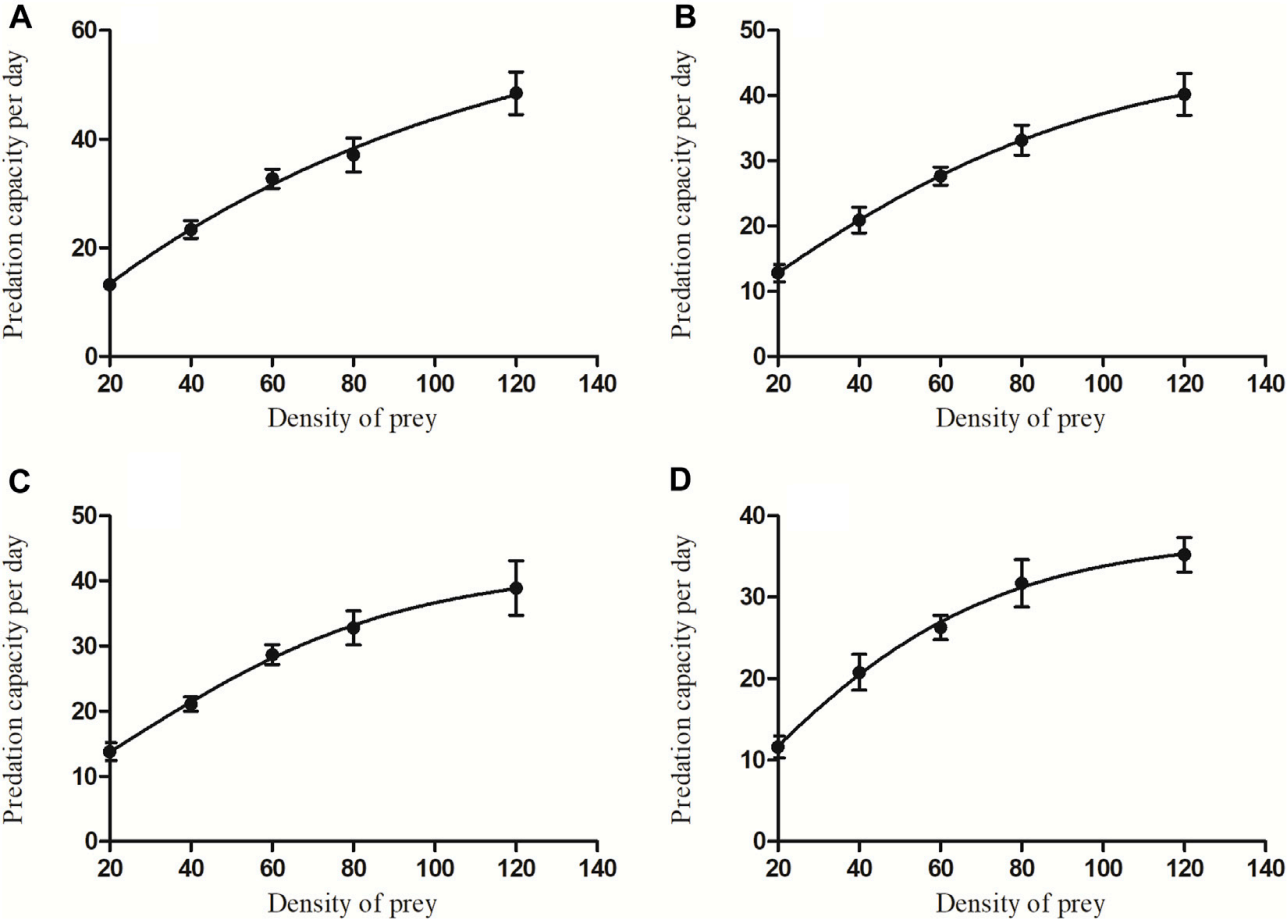
The average predation quantity of four-day-old *C. pallens* adult on *A. theae* at different developmental stages [**(A)**: egg; **(B)**: larva; **(C)**: nymph; **(D)**: adult] and different prey densities.

Predation by *C. pallens* for different densities and developmental stages of *A. theae* displayed a similar trend (Figure 3). Initially, the prey mite consumption significantly increased as the *A. theae* density gradually increased and then tended to level off (eggs: *F* = 28.206, *df* = 4.45, *p* < 0.0001; larvae: *F* = 24.568, *df* = 4.45, *p* < 0.0001; nymphs: *F* = 16.356, *df* = 4.45, *p* < 0.0001; adults: *F* = 19.881, *df* = 4.45, *p* < 0.0001).

The results showed that the functional response of adult *C. pallens* to the four developmental stages, eggs, larvae, nymphs, and adult mites, belonged to the Type II Holling model. The functional response equations and parameters are listed in Table 3. The correlation coefficient R^2^ of *A. theae* eggs, larvae, nymphs, and adults was 0.98, 0.964, 0.948, and 0.902, respectively. The Chi-square tests showed that the observational results were not significantly different from theoretic prey consumptions, with χ^2^ value of 0.072, 0.02, 0.067, and 0.163 and *p*-values of 0.999, 0.994, 0.996, and 0.993 for *A. theae* eggs, larva, nymph, and adult, respectively.

**TABLE 3:** Parameters of functional response of *C. pallens*’ predation on *A. theae*. Note: N_a-max_ = a′/T_h_ (the theoretical maximum number of prey consumed per day).

Stage	Functional response equation	*R* ^*2*^	a′	T_h_	N_a-max_ (a′/T_h_)	*X* ^*2*^	*P*
Egg	Na = 0.76N/(1 + 0.007N)	0.98	0.76	0.01	76	0.072	0.999
Larva	Na = 0.764N/(1 + 0.01N)	0.964	0.764	0.014	54.571	0.02	0.994
Nymph	Na = 0.858N/(1 + 0.016N)	0.948	0.858	0.016	53.625	0.067	0.996
Adult	Na = 0.735N/(1 + 0.011N)	0.902	0.735	0.016	45.938	0.163	0.993

## Discussion

A green, economic, and healthy tea industry requires a more environmentally friendly pest management strategy. Therefore, it is important to identify natural enemies as candidate predators for the development of biological control in tea gardens. In this study, we established a PCR-based assay for gut DNA content analyses and assessed the potential of *C. pallens* as a biological control agent for *A. theae*, a serious mite pest of tea plants. This is the first study to focus on the prey relationship between a generalist predator and a tea mite pest, providing direct evidence of *C. pallens*’ predation on *A. theae*.

We succefully obtained the prey *A. theae*-specific primers (Table 1). The 28S ribosomal RNA gene was selected to design *A. theae*-specific primers because of its abundant divergent domains and reported role in the molecular classification and identification of mites (Zhao et al., 2020). The results of the primer specificity test demonstrated its species specificity because an expected 136 bp fragment was amplified from *A. theae* DNA and not from 38 non-target species or negative controls (Supplementary Figure S1). When employ species-specific primers to trace predator-prey interactions, it is important to consider the decline in prey DNA in predator guts. Our study showed that 83.3% of the *C. pallens* tested positive when killed immediately following feeding (t = 0) in comparison to subsequent times, and the prey DNA of *A. theae* DS_50_ was calculated as 2.9 h (Figure 1). The DS_50_ value was shorter than that of *Chrysoperla plorabunda* (Fitch) preying on corn leaf aphid *Rhopalosiphum maidis* (Fitch) (3.95 h) (Greenstone et al., 2014), *H. axyridis* preying on the aphid *Eucallipterus tiliae* L. (3.1 h) (Rondoni et al., 2015), and the mirid bug *Apolygus lucorum* (4.4 h) (Li et al., 2017) but was longer than that of *H. axyridis* preying on ladybirds, *Adalia bipunctata* L. (1.2 h) (Rondoni et al., 2015). A higher DS_50_ value usually means that the prey is more likely to be detectable from its predators (Greenstone et al., 2014). To the best of our knowledge, our study is the first to determine the DS_50_ values for *C. pallens*’ predation on a tea mite pest.

To evaluate the potential of *C. pallens* as a biological control agent for *A. theae*, it is necessary to determine whether *C. pallens* can prey on *A. theae*, particularly in the field. Owing to the small body size and cryptic occurrence of *A. theae* (Yin et al., 2003; Li et al., 2021), it is difficult to estimate prey relationships through direct observation. Our results of DNA gut-content analyses of field-collected samples showed that 23.53% of *C. pallens* contained *A. theae* DNA. This positive detectability provides direct evidence of the existence of a trophic prey relationship between *C. pallens* and *A. theae* in the field. Former research has revealed that temperature has a great influence on the egg hatching, the survival rate of larvae and pupae, and the lifespan of adult *C. pallens* (Zhao, 1988), and persistent high temperature during *C. pallens* collection may be responsible for the low number of lacewing samples. In addition, temperature is reportedly associated with *C. pallens*’ predation (Feng et al., 2021), which could account for the different detection rates between laboratory and field condition.

Functional responses are important indicators of the effectiveness of natural enemies in suppressing pest populations (Fantinou et al., 2012; Ganjisaffar and Perring, 2015). Our results showed that the functional predatory responses of *C. pallens* to *A. theae* fit the Type II Holling model. When prey density increased, the net prey consumption by *C. pallens* increased until a plateau was established. The correlation coefficient R^2^ of *A. theae* eggs, larva, nymph and adult ranged from 0.902 to 0.98, and χ^2^ value of the Chi-square tests ranged from 0.02 to 0.163 (Figure 3; Table 3). This functional response resembles the models in which *C. pallens* preys on insect pests, including the aphids *Myzus persicae* and *Aphis nerii* (Zhao et al., 2008) and larvae of the fall armyworm *Spodoptera frugiperda* (Cao et al., 2020).

It has been discovered that the two crucial parameters, instant attack rate (a’) and the time required to consume on a prey (T_h_), accurately characterize most of the variation in prey and predator interactions. The maximal prey consumption estimate (=a’/T_h_) provided the opportunity to determine the ideal predator-target pest ratio, which can be beneficial in streamlining the utilization of inoculative releases. Our study showed an initial attack rate a’ of 0.735–0.858 and a handling time T_h_ approximate at 0.01, and the maximal prey consumption estimate (=a’/T_h_) of *C. pallens* preying on *A. theae* different developmental stages ranged from 45.938 to 76 (Table 3). These results showed a higher instant attack rate and shorter consequent handling time than those of predation of *C. pallens* larvae on *Bemisia tabaci* (Wang et al., 2016), and predation of *C. pallens* adult on 2nd- and 3rd-instar *S*. *frugiperda* larvae (Cao et al., 2020).

In addition, *C. pallens* displayed no preferential feeding for the different developmental stages of *A. theae* (Figure 2), suggesting its common predation potential on all *A. theae* life stages. While these findings offer a preliminary glimpse into the potential of *C. pallens* as a biological control agent for *A. theae* in tea gardens, comprehensive research and extensive field trials are essential to definitively establish its suitability and effectiveness in this role since the predation ability of predator is always influenced by climate conditions, prey density, and its own density (Desneux et al., 2006; Talebi et al., 2022).

## Data Availability

The datasets presented in this study can be found in online repositories. The names of the repository/repositories and accession number(s) can be found in the article/Supplementary Material.

## References

[B1] Abdrabou S. (2008). Evaluation of the green lacewing, *Chrysoperla carnea* (Stephens) (Neuroptera; Chrysopidae) against aphids on different crops. J. Biol. Control 22 (2), 299–310. 10.18311/jbc/2008/3763

[B2] Canard M. Volkovich T. A. (2001). “Outlines of lacewing development,” in Lacewings in the crop environment. Editors McEwen P. K. New R. Whittington A. E. (Cambridge, MA, USA: Cambridge University Press), 130–154. 10.1017/CBO9780511666117.008

[B3] Cao W. Zhang T. Yang H. Xu Z. Gu J. Chen H. (2020). Evaluation of predatory function of *Chrysopa pallens* to larvae of fall armyworm *Spodoptera frugiperda* . J. Plant Prot. 47 (4), 839–844. 10.13802/j.cnki.zwbhxb.2020.2020821

[B4] Desneux N. O’neil R. J. Yoo H. J. S. (2006). Suppression of population growth of the soybean aphid, *Aphis Glycines* Matsumura, by predators: the identification of a key predator and the effects of prey dispersion, predator abundance, and temperature. Environ. Entomol. 35 (5), 1342–1349. 10.1603/0046-225X(2006)35[1342:SOPGOT]2.0.CO;2

[B5] Fantinou A. A. Baxevani A. Drizou F. Labropoulos P. Perdikis D. Papadoulis G. (2012). Consumption rate, functional response and preference of the predaceous mite *Iphiseius degenerans* to *Tetranychus urticae* and *Eutetranychus orientalis* . Exp. App. Carol. 58 (2), 133–144. 10.1007/s10493-012-9557-6 22527836

[B6] Feng W. Hu C. Zhu Q. (2021). Effects of temperature and space on predation of *Dasumeira pyri* by *Chrysopa pallens* . Shandong Agric. Sci. 53 (9), 111–115. 10.14083/j.issn.1001-4942.2021.09.019

[B7] Ganjisaffar F. Perring T. (2015). Prey stage preference and functional response of the predatory mite *Galendromus flumenis* to Oligonychus pratensis. Biol. Control 82, 40–45. 10.1016/j.biocontrol.2014.12.004

[B8] Greenstone M. H. Payton M. E. Weber D. C. Simmons A. M. (2014). The detectability half-life in arthropod predator-prey research: what it is, why we need it, how to measure it, and how to use it. Mol. Ecol. 23 (15), 3799–3813. 10.1111/mec.12552 24303920

[B9] Greenstone M. H. Rowley D. L. Weber D. C. Payton M. E. Hawthorne D. J. (2007). Feeding mode and prey detectability half-lives in molecular gut-content analysis: an example with two predators of the Colorado potato beetle. Bull. Entomol. Res. 97 (2), 201–209. 10.1017/s000748530700497x 17411483

[B10] Guo J. Y. Wan F. H. Dong L. Lövei G. L. Han Z. J. (2008). Tri-trophic interactions between bt cotton, the herbivore *Aphis gossypii* glover (Homoptera: Aphididae), and the predator *Chrysopa pallens* (rambur) (neuroptera: Chrysopidae). Environ. Entomol. 37 (1), 263–270. 10.1603/0046-225x(2008)37[263:tibbct]2.0.co;2 18348819

[B11] Hall T. A. (1999). BioEdit: a user-friendly biological sequence alignment editor and analysis program for Windows 95/98/NT. Nucleic Acids Symp. Ser. 41 (41), 95–98. 10.1021/bk-1999-0734.ch008

[B12] He P. Mang D. Z. Wang H. Wang M. M. Ma Y. F. Wang J. (2020). Molecular characterization and functional analysis of a novel candidate of cuticle carboxylesterase in *Spodoptera exigua* degradating sex pheromones and plant volatile esters. Pestic. Biochem. Phys. 163, 227–234. 10.1016/j.pestbp.2019.11.022 31973861

[B13] He P. Zhang Y. F. Hong D. Y. Wang J. Wang X. L. Zuo L. H. (2017). A reference gene set for sex pheromone biosynthesis and degradation genes from the diamondback moth, *Plutella xylostella*, based on genome and transcriptome digital gene expression analyses. BMC Genomics 18 (1), 219. 10.1186/s12864-017-3592-y 28249567 PMC5333385

[B14] Holling C. S. (1959). Some characteristics of simple types of predation and parasitism. Can. Entomol. 91 (7), 385–398. 10.4039/Ent91385-7

[B15] King R. A. Read D. S. Traugott M. Symondson W. O. (2008). Molecular analysis of predation: a review of best practice for DNA-based approaches. Mol. Ecol. 17 (4), 947–963. 10.1111/j.1365-294X.2007.03613.x 18208490

[B16] Kitto G. B. (1983). Biological control of insect pests. Isozymes Curr. Top. Biol. Med. Res. 11, 197–211. 6642987

[B17] Kunkel B. A. Cottrell T. E. (2007). Oviposition response of green lacewings (Neuroptera: Chrysopidae) to aphids (Hemiptera: Aphididae) and potential attractants on pecan. Environ. Entomol. 36 (3), 577–583. 10.1603/0046-225x(2007)36[577:orogln]2.0.co;2 17540067

[B18] Lai Y. Liu X. (2020). The natural enemy species of Chrysopidae from China and their applications in biological control: a review. J. Plant Prot. Res. 47 (6), 1169–1187. 10.13802/j.cnki.zwbhxb.2020.2020250

[B19] Lalitha S. (2000). Primer premier 5. Biotech Softw. Internet Rep. 1 (6), 270–272. 10.1089/152791600459894

[B20] Li H. Cui H. Mao Y. Zheng X. Huang H. Zhao Y. (2021). Pests on tea leaves—pink mite (*Acaphylla theae* Watt). China Tea 43 (1), 37–39. 10.3969/j.issn.1000-3150.2021.01.006

[B21] Li J. Yang F. Wang Q. Pan H. Yuan H. Lu Y. (2017). Predation by generalist arthropod predators on *Apolygus lucorum* (Hemiptera: Miridae): molecular gut-content analysis and field-cage assessment. Pest Manag. Sci. 73 (3), 628–635. 10.1002/ps.4346 27349598

[B22] Liao Y. Zhou X. Zeng L. (2022). How does tea (*Camellia sinensis*) produce specialized metabolites which determine its unique quality and function: a review. Crit. Rev. Food Sci. Nutr. 62 (14), 3751–3767. 10.1080/10408398.2020.1868970 33401945

[B23] Liu S. Wang S. Liu B. M. Zhou C. Q. Zhang F. (2011). The predation function response and predatory behavior observation of *Chrysopa pallens* larva to *Bemisia tabaci* . Sci. Agric. Sin. 44 (6), 1136–1145. 10.3864/j.issn.0578-1752.2011.06.008

[B24] Rondoni G. Athey K. J. Harwood J. D. Conti E. Ricci C. Obrycki J. J. (2015). Development and application of molecular gut-content analysis to detect aphid and coccinellid predation by *Harmonia axyridis* (Coleoptera: coccinellidae) in Italy. Insect Sci. 22 (6), 719–730. 10.1111/1744-7917.12165 25164698

[B25] Sarkar S. C. Wang E. Zhang Z. Wu S. Lei Z. (2019). Laboratory and glasshouse evaluation of the green lacewing, *Chrysopa pallens* (Neuroptera: Chrysopidae) against the western fower thrips, *Frankliniella occidentalis* (Thysanoptera: thripidae). Appl. Entomol. Zool. 54, 115–121. 10.1007/s13355-018-0601-9

[B26] Su Y. Ren X. Ma X. Wang D. Hu H. Song X. (2022). Evaluation of the toxicity and sublethal effects of acetamiprid and dinotefuran on the predator *Chrysopa pallens* (Rambur) (Neuroptera: Chrysopidae). Toxics 10 (6), 309. 10.3390/toxics10060309 35736917 PMC9228657

[B27] Sun L. Yi W. Zhao C. Dong X. (2013). Predatory capacity of *Chrysopa pallens* (Rambur) to three species of aphids. Plant Prot. 39 (5), 153–157. 10.3969/j.issn.0529-1542.2013.05.023

[B28] Symondson W. O. (2002). Molecular identification of prey in predator diets. Mol. Ecol. 11 (4), 627–641. 10.1046/j.1365-294x.2002.01471.x 11972753

[B29] Talebi A. A. Kazemi M. Rezaei M. Mirhosseini M. A. Moharramipour S. (2022). Host stage preference and temperature-dependent functional response of *Diaeretiella rapae* (Hymenoptera: braconidae) on *Schizaphis graminum* (Hemiptera: Aphididae). Int. J. Trop. Insect Sci. 42 (1), 415–424. 10.1007/s42690-021-00558-9

[B30] Tang T. C. Zhang Y. Li C. J. Cao X. R. Chen Z. Z. Xu Y. Y. (2018). Predatory responses of *Chrysoperla sinica* (Tjeder) and *Chrysopa pallens* larvae to *Aleurocan spinfetus* (Quaintance) nymphs. Chin. J. Appl. Entomol. 55 (2), 217–222. 10.7679/j.issn.2095-1353.2018.030

[B31] Tong S. M. Feng M. G. (2016). Laboratory and field evaluations of camptothecin sodium salt against phytophagous mites. Pest Manag. Sci. 72 (3), 629–636. 10.1002/ps.4033 25924840

[B32] Traugott M. Kamenova S. Ruess L. Seeber J. Plantegenest M. (2013). “Chapter three-empirically characterising trophic networks: what emerging DNA-based methods, stable isotope and fatty acid analyses can offer,” in Advances in ecological research. Editors Woodward G. Bohan D. A., 49, 177–224. 10.1016/B978-0-12-420002-9.00003-2

[B33] Wang J. Li S. Yang J. Guo M. Dai H. Ramirez-Romero R. (2021). The fitness of mass rearing food on the establishment of *Chrysopa pallens* in a banker plant system under fluctuating temperature conditions. Insects 12 (11), 1014. 10.3390/insects12111014 34821814 PMC8619634

[B34] Wang Q. Li Y. Wang Q. Sun L. Zhang Y. (2022a). The *Adelphocoris lineolatus* OBP4: support for evolutionary and functional divergence of a mirid pheromone-binding protein from that found in lepidopteran moths. Insect Sci. 29 (1), 151–161. 10.1111/1744-7917.12919 33890408

[B35] Wang Q. Wang Q. Li H. Sun L. Zhang D. Zhang Y. (2020). Sensilla localization and sex pheromone recognition of odorant binding protein OBP4 in the mirid plant bug *Adelphocoris lineolatus* (Goeze). J. Insect Physiol. 121, 104012. 10.1016/j.jinsphys.2020.104012 31911184

[B36] Wang Q. Zhang R. Wang M. Zhang L. Shi C. M. Li J. (2022b). The first chromosome-level genome assembly of a green lacewing *Chrysopa pallens* and its implication for biological control. Mol. Ecol. Res. 22 (2), 755–767. 10.1111/1755-0998.13503 PMC9292380 34549894

[B37] Wang R. Wang S. Qu C. Li J. Chen Z. Zhang F. (2016). The predatory functional response and searching effect of *Chrysopa pallens* larvae to *Bemisia tabaci* eggs on different host plants. J. Plant Prot. 43 (1), 149–154. 10.13802/j.cnki.zwbhxb.2016.01.022

[B38] Wang S. X. Di N. Chen X. Zhang F. Biondi A. Desneux N. (2019). Life history and functional response to prey density of the flower bug *Orius sauteri* attacking the fungivorous sciarid fly *Lycoriella pleuroti* . J. Pest Sci. 92 (2), 715–722. 10.1007/s10340-018-1032-7

[B39] Yan Y. Zhang Y. Tu X. Wang Q. Li Y. Li H. (2020). Functional characterization of a binding protein for Type-II sex pheromones in the tea geometrid moth *Ectropis obliqua* Prout. Pestic. Biochem. Phys. 165, 104542. 10.1016/j.pestbp.2020.02.008 32359552

[B40] Yang C. S. Lambert J. D. Sang S. (2009). Antioxidative and anti-carcinogenic activities of tea polyphenols. Arch. Toxicol. 83 (1), 11–21. 10.1007/s00204-008-0372-0 19002670 PMC2820244

[B41] Yang F. Yao Z. W. Zhu Y. Wu Y. K. Liu L. T. Liu B. (2020). A molecular detection approach for assessing wheat aphid-parasitoid food webs in northern China. Entomol. Gen. 40, 273–284. 10.1127/entomologia/2020/1009

[B42] Yao M. Guo H. Wang X. Xiao Q. Chen L. (2008). The variation of resistance to pink mite among tea germplasm and screening of high-resistant and excellent landraces from Wuyishan region in Fujian. Chin. Agr. Sci. Bull. 24 (9), 127–131. 10.11924/j.issn.1000-6850.2008-0135

[B43] Ye G. Y. Xiao Q. Chen M. Chen X. X. Yuan Z. J. Stanley D. W. (2014). Tea: biological control of insect and mite pests in China. Biol. Control 68, 73–91. 10.1016/j.biocontrol.2013.06.013

[B44] Ye L. Zhong W. Xu B. Xia X. Lai Z. (2021). The agriculture-tourism integration to promote the rural revitalization: taking tea industry—agriculture—tourism in Lishui city as an example. Asian Agr. Res. 13 (12), 23–25. 10.22004/ag.econ.317735

[B45] Yin K. S. Tang M. J. Xiong X. P. Chen H. C. (2003). Studies on the effect of ecological factors on the population dynamics of tea pink mite (*Acaphylla theae*). J. Tea Sci. 23, 53–57. 10.3969/j.issn.1000-369X.2003.z1.009

[B46] Zhang S. Liu F. Yang B. Liu Y. Wang G. R. (2023). Functional characterization of sex pheromone receptors in *Spodoptera frugiperda, S. exigua*, and *S. litura* moths. Insect Sci. 30 (2), 305–320. 10.1111/1744-7917.13098 35932282

[B47] Zhao J. Z. (1988). Studies on the bionomics of *Chrysopa septempunctata* wesmoer. Acta Phytophylacica Sin. 15 (2), 123–127. 10.13802/j.cnki.zwbhxb.1988.02.012

[B48] Zhao Q. Chen J. Liu F. X. Xiao W. F. Peng Y. (2008). Predation of *Chrysopa pallens* on *Myzus persicae* and *Aphis nerii* . J. Environ. Entomol. 30 (3), 220–223. 10.1016/S1005-9040(08)60003-3

[B49] Zhao Y. Zhang W. Y. Wang R. L. Niu D. L. (2020). Divergent domains of 28S ribosomal RNA gene: DNA barcodes for molecular classification and identification of mites. Parasite. Vector. 13 (1), 251. 10.1186/s13071-020-04124-z PMC7222323 32404192

