# Supplementary material for: Predation evaluation of the green lacewing, Chrysopa pallens on the pink tea mite pest, Acaphylla theae (Watt) (Acarina: Eriophyidae)
Source: Front Physiol. 2023 Dec 12;14:1307579. doi: 10.3389/fphys.2023.1307579 (PMC10751929; doi:10.3389/fphys.2023.1307579)
Supplement: Supplementary file 1 [file Image1.pdf]

Supplementary Material：

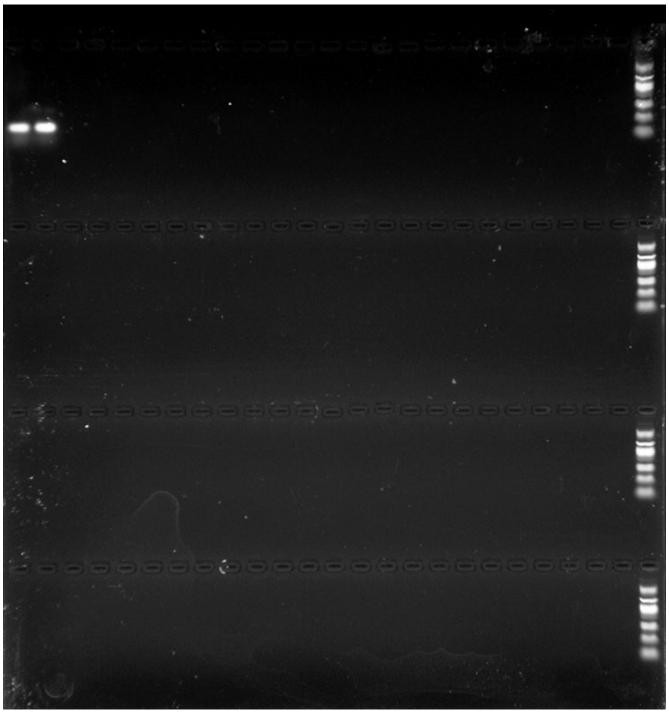

Figure S1 PCR amplifications of the extracted DNA samples using *A. theae*-specific primers targeting the 28S rRNA gene. Each sample includes two biological replicates. Lanes 1 and 2, *A. theae*; lanes 3 and 4, negative controls; lanes 5–80, 38 non-targeted invertebrates similar to the list in Table 2. M is a 500 bp DNA ladder.
